# Spectral Signature Generalization and Expansion Can Improve the Accuracy of Satellite Image Classification

**DOI:** 10.1371/journal.pone.0010516

**Published:** 2010-05-06

**Authors:** Alice G. Laborte, Aileen A. Maunahan, Robert J. Hijmans

**Affiliations:** International Rice Research Institute, Los Baños, Laguna, Philippines; Purdue University, United States of America

## Abstract

Conventional supervised classification of satellite images uses a single multi-band image and coincident ground observations to construct spectral signatures of land cover classes. We compared this approach with three alternatives that derive signatures from multiple images and time periods: (1) *signature generalization*: spectral signatures are derived from multiple images within one season, but perhaps from different years; (2) *signature expansion*: spectral signatures are created with data from images acquired during different seasons of the same year; and (3) combinations of expansion and generalization. Using data for northern Laos, we assessed the quality of these different signatures to (a) classify the images used to derive the signature, and (b) for use in temporal signature extension, i.e., applying a signature obtained from data of one or several years to images from other years. When applying signatures to the images they were derived from, signature expansion improved accuracy relative to the conventional method, and variability in accuracy declined markedly. In contrast, signature generalization did not improve classification. When applying signatures to images of other years (temporal extension), the conventional method, using a signature derived from a single image, resulted in very low classification accuracy. Signature expansion also performed poorly but multi-year signature generalization performed much better and this appears to be a promising approach in the temporal extension of spectral signatures for satellite image classification.

## Introduction

Satellite remote sensing programs have produced an archive of images of the earth that are becoming an increasingly valuable source of data for the study of land cover and land use change. The foremost example is the Landsat program, which has been in operation since 1972. The entire Landsat archive has become freely available, allowing public access to time-series data for most parts of the world. Interpretation of these images, however, remains a challenge.

Conventional supervised image classification relies on *training data* (sites for which there are direct observations of land cover) that coincide temporally with the images used. Training data and the multi-spectral satellite data for the same sites are used in multivariate statistical algorithms to create a predictive model, referred to as “spectral signatures”, that is used to classify the satellite image into land cover classes. Training data, however, are usually not available for the majority of images in a time series, and can, in many cases, no longer be easily obtained for older images.

One approach to overcome this problem of missing training data is using visual interpretation, but this is difficult, time-consuming [1], and possibly very subjective. An alternative approach is to use a signature derived from training data and a matching image from another period and apply this to the images for which no training data are available. Such *signature extension* (referred to as *signature generalization* by [2], [3]) has been used to classify images by applying signatures obtained from a different domain, whether location, time period, or sensor [4], [5]. Studies that date back to the 1970s have explored signature extension for Landsat Multi-Spectral Scanner (MSS) images [4], [6]. More recently, this approach has been re-examined in response to advances in atmospheric correction and the need to monitor large areas efficiently [2], [3], [5].

The accuracy of spatial signature extension, which uses signatures derived from training sites from one region to classify images from another region, has been found to deteriorate with distance between the regions [3], [5]. In one study, a distance of 1500 to 2000 km between the signature source and the image to be classified reduced the accuracy by half compared with a distance of 500 km [5]. That study also reported poorer accuracy in signature extension in the north–south than in the east–west direction due to the larger change in vegetation in the north–south direction [5].

Temporal signature extension has yielded better results than spatial signature extension [2], particularly when variation across years is reduced with radiometric normalization (or rectification, [7]) [5], but the general validity of the conventional approach to signature extension has not been investigated much, and alternative approaches, such as combining data from several images, have not been considered.

A potential problem in temporal signature extension is that time-series of archived high-quality (cloud-free) images are rarely available exactly for the same time periods across years. One could apply a signature to an image for another year and time of the year, but this may further diminish classification accuracy because reflectance of some land cover classes changes throughout the year. This type of cyclic variation is particularly strong for annual crops, and in areas where vegetation growth is reduced in cold or dry seasons. However, cyclic variation also presents an opportunity. By using images from different time periods, classes may become easier to distinguish [8]. Thus the use of multiple images per year could improve classification accuracy.

In this paper, we compare different methods of combining satellite images to derive improved signatures. We first evaluate whether such signatures improve the classification of the images used in their construction. However, the principal goal of this paper is to evaluate whether signatures derived from combined images perform better when used in signature extension, that is, when they are applied to classify images for other years.

## Materials and Methods

### Study area

**Figure 1:**
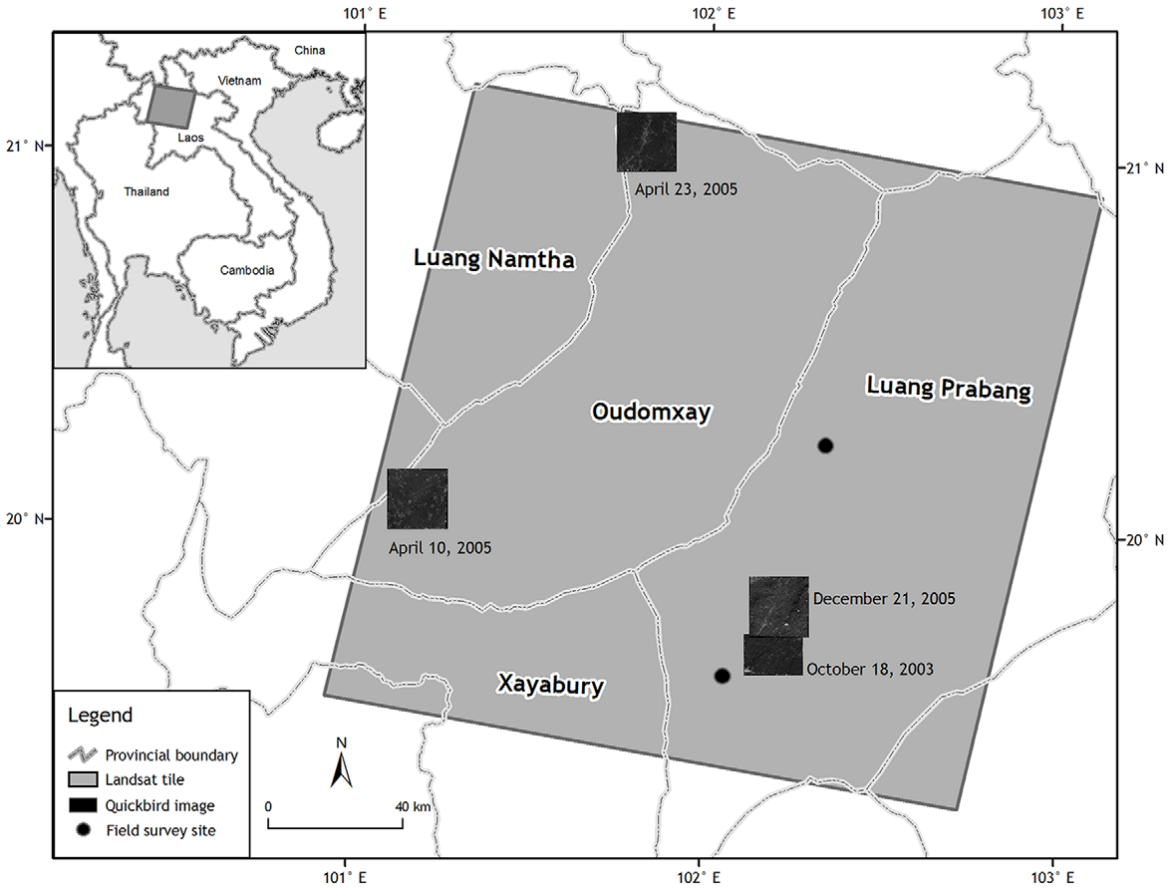
Study area and sources of training and test data. Small black insets are Quickbird images, black dots are approximate areas of field surveys.

The study area, in northern Laos, is covered by the Landsat Worldwide Reference System (WRS 2) path 129 row 46 (Figure 1), and it comprises about 34000 km^2^. The area is mountainous, with elevations ranging from 274 to 1810 m. The rainy season is from May to October, with an average annual rainfall of about 1400 mm. A typical landscape in this area consists of patches cleared for cropping, recent and old fallow fields, and dense forests, which are usually located at higher elevations and on very steep slopes. There is land under permanent cultivation in the valleys. Rice is the dominant crop. It is usually planted in late May or early June and harvested in October to November. Other crops grown on the sloping fields include sesame and maize. On land used for shifting cultivation, the vegetation is usually cut in January or February and burned in March or April.

### Landsat data and pre-processing

We acquired all available near-to-cloud-free Landsat Enhanced Thematic Mapper Plus (ETM+) images from 2003 to 2006 for the study area (Table 1). Because of the failure of the Scan Line Corrector of Landsat 7, images collected after July 14, 2003, have gaps. Interpolated values in these gaps were not used in our assessments. All images were projected to UTM zone 48 (WGS 1984 datum) using a nearest neighbor algorithm with a cell size of 28.5 m. They were all co-registered to the November 2000 image using 50 tie points. A first order transformation using nearest neighbor resampling was used. The average root mean square error of all transformations was less than a pixel (26 m). We converted the images to exo-atmospheric reflectance values to correct for illumination. The multivariate alteration detection (MAD) transformation was used to obtain invariant pixels for automatic relative radiometric normalization of the time-series images [9]. With this method, no decision thresholds nor subjective criteria for defining pseudo invariant features need to be defined as the method automatically selects the features that have not changed. Although a number of techniques for absolute atmospheric correction have been proposed, meteorological or atmospheric input data are usually not available and alternatives to such complex and sophisticated methods have been found to be effective [10].

**Table 1:** Landsat images used in the study^a^.

Year	Month and day				
2000	November 2				
2002	February 9				
2003	February 28	November 11			
2004	January 30	March 2	October 28	November 13	
2005	February 1	February 17	March 21	September 13	December 18
2006	January 19	March 8	August 15	November 3	

a The 2000 and 2002 images were only used in the relative radiometric normalization.

Images from the same season were normalized using the image with the “best” radiometric quality determined through visual inspection. All images from the first quarter (January to March) were normalized with the February 9, 2002 image and all other images were normalized with the November 2, 2000 image. Because the area is mountainous, topographic correction was done using the 90-m digital elevation data from the shuttle radar topography mission (SRTM) resampled to 28.5 m using bilinear interpolation. For each satellite image, areas with clouds, cloud and mountain shadows, and water bodies were removed from the analysis.

### Image classification

**Figure 2:**
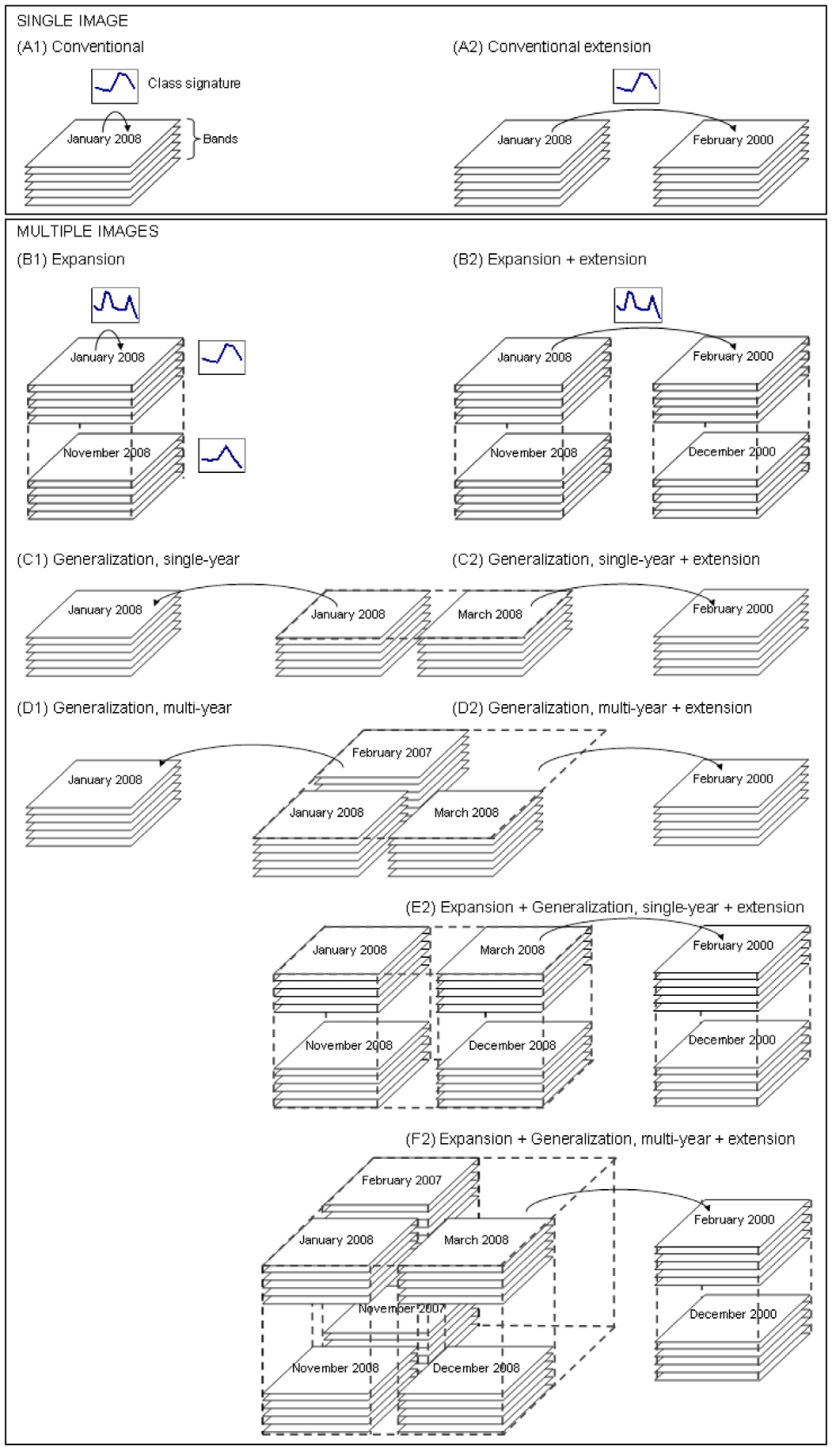
Methods used to create land cover class spectral signatures and their use in classification. Methods: A (conventional), B (expansion), C (single-year generalization), D (multi-year generalization), E (single-year expansion + generalization), F (multi-year expansion + generalization); and 1 (no signature extension), 2 (signature extension).

Figure 2 summarizes the approaches we considered to obtain spectral signatures for land cover classes. Signatures can be derived in the *conventional* manner, i.e., from a single image (A), or by using multiple images (B–F). *Signature expansion* (B) consists of integrating two (or more) images from different seasons within a single year. The images are “stacked” and treated as additional predictor variables (spectral bands), and training data for the same year are used. In *signature generalization* (C–D), additional images are treated as additional observations, i.e., the number of predictor variables (bands) remains the same. By using more than one image, the overall signal to noise ratio might be higher than that associated with either of the single images. Figure 2E–F illustrates combinations of signature expansion and generalization. In all cases, signatures derived from multiple images can be used for classifying land cover for the period covered by the images used. They can also be used for *temporal extension*, i.e., to classify images for a different time period.

We have attempted to select clear and unambiguous names for the different approaches we considered. Note, however, that some authors use the term *signature generalization* [2], [3] for what we and others refer to as *signature extension* [5]. We use *generalization* to refer to the creation of what is likely to be a more general signature for a given season because it is derived from multi-date images from the same season, rather than to refer to its application to a different temporal or spatial domain (without necessarily knowing whether the signature is generally valid for those domains).

We used maximum likelihood estimation, which is the most commonly used supervised classification method in the field, as implemented in the ENVI software. In addition to the Landsat data, we used slope, calculated from the elevation data, in the model fitting. After each classification, we performed a majority filter over a 3×3 neighborhood to remove speckles.

We used the following broad land cover classes: 1) dense and secondary forest or old fallow fields with trees; 2) areas with shrubs and grasses such as in recently fallowed fields; 3) agricultural land; and 4) built-up and barren land. In the training and classifications, areas under permanent and shifting cultivation were treated as separate classes. Because the signatures of permanent and shifting cultivation are similar, except at the start of the year when vegetation is sometimes more dense for shifting cultivation (i.e., the vegetation has not been cleared yet), the two land cover classes were merged post-classification under “agricultural land” and reported as such.

Each of the 15 single-date images from 2003 to 2006 (Table 1) was classified using the signature derived from training data obtained for the same year and from the same image (Figure 2 (A1)). In signature expansion, all possible combinations of images from different seasons in a single year were used (B1). The seasons considered in this study were before (January to March), during (August to October), and after (November to December) the rainy season. Henceforth, we refer to images from these three periods as early, middle, and late images.

We applied two types of signature extension: using a single image (A2) and using multiple images (B2, C2, D2, E2, F2). Temporal signature extension involving a single image is the conventional approach [2], [3], [5], in which signatures are applied to the classification of another image from the same season in a different year.

In signature expansion, images from two seasons in one year – one early and one late image – were used to create the signatures. These signatures were applied to two images (also early and late seasons) from a different year. Because we had very few near-to-cloud-free images for the rainy season, combinations of images with this season were not considered in signature extension. Two types of signature generalization were tested: using images from a single year (C) and using images for multiple years (D). The derived signatures were used to classify images for the same season in all years.

We combined expansion and generalization by first applying generalization to early or late images from the same year and then combining the early and late generalizations from one year (E2) or from several years (F2).

In all evaluations of the accuracy of signature extensions, signatures derived from training data on the combined images was used to classify other images for a year not included in the generalization. The total number of classifications was 329.

### Training and test data

We used two sources of data for training the classifier and for testing the result of the classification: very high resolution satellite imagery and a field survey. We used four QuickBird satellite images (<3 m resolution) from the following dates: October 18, 2003; April 10, 2005; April 23, 2005; and December 21, 2005 (Figure 1). Sites pertaining to the target land classes were located using these images. Selection of sites for each land cover class, and for each year, was done using purposive sampling. For each land cover class, training/test sites were selected such that the land cover was homogenous within a 30 m radius. Each training/test site corresponded to one pixel on the image. For 2004 and 2006, additional sites were located based on the 2003 and 2005 land cover classes, visual interpretation of the dry-season satellite images, and simple decision rules (in particular, the presence or absence of vegetation from January to March, during which time vegetation is cleared for shifting cultivation).

In addition, a field survey was conducted in November 2006 in the province of Luang Prabang. We were able to classify less accessible sites by taking photographs of the landscape and recording the locations and directions in which the photographs were taken using a global positioning system (GPS) receiver and a compass. For these sites, coordinates were estimated by locating the photographer's position and matching the landscape photo with a 3D rendering of the November 3, 2006 Landsat image.

The number of sites obtained was 811 for 2003, 762 for 2004, 932 for 2005, and 466 for 2006. These sites were divided randomly and half for each land class were used as training sites and the other half for evaluating the accuracy of the classifications.

Although the set of test sites was held constant for each year, the specific sites that could be used were not the same for all classifications in the comparison of single and multi-date stacks from different seasons. This is due to the gaps in the Landsat images and large masked-out areas due to clouds and shadows, particularly for images taken during the rainy season. Using exactly the same set across comparisons would have greatly reduced the number of sites available. For 2004 and 2005, only 59 and 58 test sites, respectively, would have remained. However, in the evaluation of different methods of signature extension, we used the same test data for each image classification across different methods.

The accuracy of each classification was assessed by calculating the *Kappa* statistic, a common measure of classification accuracy that accounts for the extent to which correctly classified values in a confusion matrix are due to actual agreement and not to chance [11]. We used the Wilcoxon rank sum test, a nonparametric test, to compare the classification accuracy values resulting from the different methods considered.

## Results

### Classification without extension

Kappa values (n = 15) for single image classification varied between 0.49 and 0.83, and the median was 0.64 (Table 2). The classification accuracy of 2003 images was consistently higher than that of images for other years. No single month or season, however, consistently had the highest image classification accuracy across years. For 2003 and 2004, Kappa was highest for the November images and lowest for the February and March images. For 2005, however, the March image had the highest Kappa, whereas, for 2006, the December image had the lowest.

**Table 2:** Comparison of accuracy of classifications using single-date and stacks of images from different seasons in a year, 2003–06^a^ (Kappa).

Image(s) used in the classification			2003	2004	2005^b^	2006
Single-date image (conventional classification, A1)						
January				0.57		0.71
February			0.78		0.55	
					0.57	
March				0.49	0.69	0.71
August						0.66
September					0.63	
October				0.52		
November			0.83	0.69		0.63
December					0.64	
Combinations of images from different seasons (expansion, B1)						
January	August					0.73
January	October			0.61		
January	November			**0.71**		0.73
February	September				0.68	
					0.66	
February	November		**0.84**			
February	December				0.71	
					0.73	
March	August					0.73
March	September				0.67	
March	October			0.61		
March	November			0.62		0.71
March	December				0.76	
August	November					0.77
October	November			0.58		
September	December				0.73	
January	August	November				**0.79**
January	October	November		0.70		
February	September	December			**0.81**	
					0.75	
March	August	November				0.74
March	September	December			0.69	
March	October	November		0.67		

a Values in bold are highest Kappa values for the year.

b There are two February images in 2005: February 1 and 17.

#### Signature expansion

For all the years considered (Table 1), the highest accuracies were obtained by classifications using combinations of images from different seasons (signature expansion, B1) and not with a single-date (A1) image. For 2003 and 2004, the classification using images from two seasons had the highest accuracy: February and November in 2003, and January and November in 2004. However, the improvement over the best single-image classifications was negligible with Kappa increasing only 0.01 to 0.02. For 2005 and 2006, accuracy was highest for classifications using images for all three seasons. In these years, signature expansion increased the accuracy of the classifications, relative to the best single-image classification, with 11% (2005) and 17% (2006) (Table 2).

**Figure 3:**
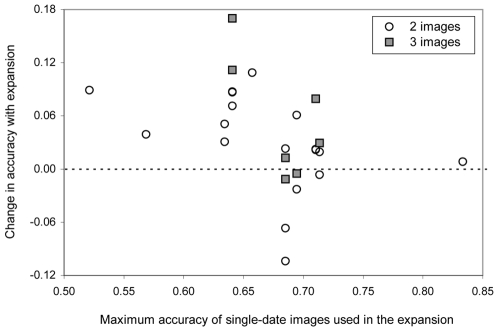
Change in accuracy (Kappa) from signature expansion as compared to single-image classification.

In 76% of the cases, accuracy was higher with signature expansion than with the use of single-date images (Table 2 and Figure 3). At lower classification accuracies of single-date images (<0.65), the increase in accuracy due to expansion ranged from 0.03 to 0.17. The two cases in which Kappa values from signature expansion declined slightly by at least 0.07 resulted from combining two images (March-November and October-November 2004), one of which has a relatively high accuracy (0.69 for November) and the other has low accuracy (≤0.52 for March and October).

**Figure 4:**
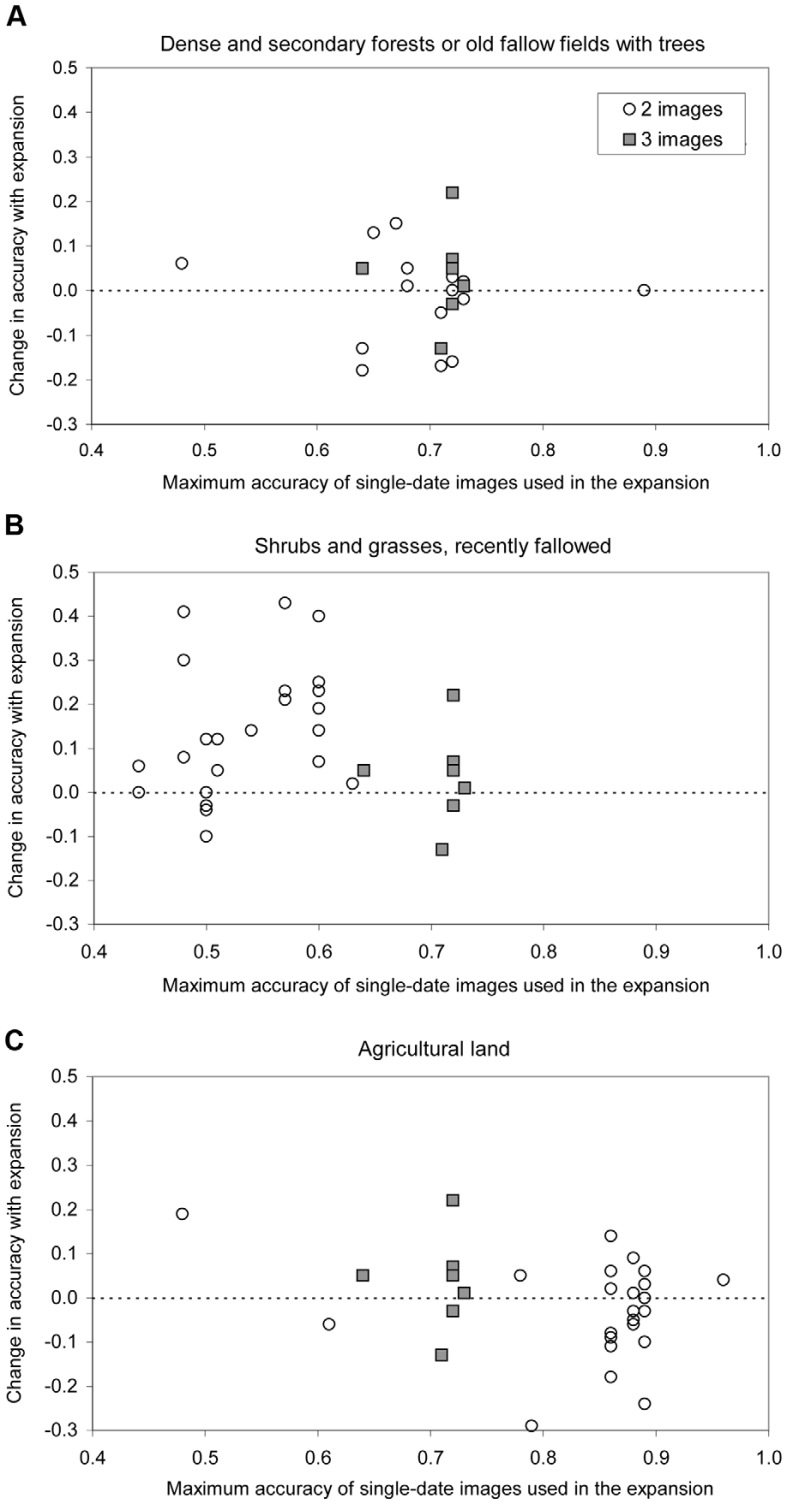
Change in accuracy resulting from signature expansion. Dense and secondary forests or old fallow trees (A), shrubs and grasses, recently fallowed (B) and agricultural land (C) are the three most common land cover classes considered in this study.

The change in accuracy when comparing classification using signature expansion with classification with single-date signatures depended on the land cover class (Figure 4). For the forest and old fallow class, 60% of the cases had a higher accuracy, whereas for shrubs and grasses 80% of the cases have a higher accuracy with signature expansion. For agricultural land, however, only about half of the cases have a higher accuracy with signature expansion.

**Figure 5:**
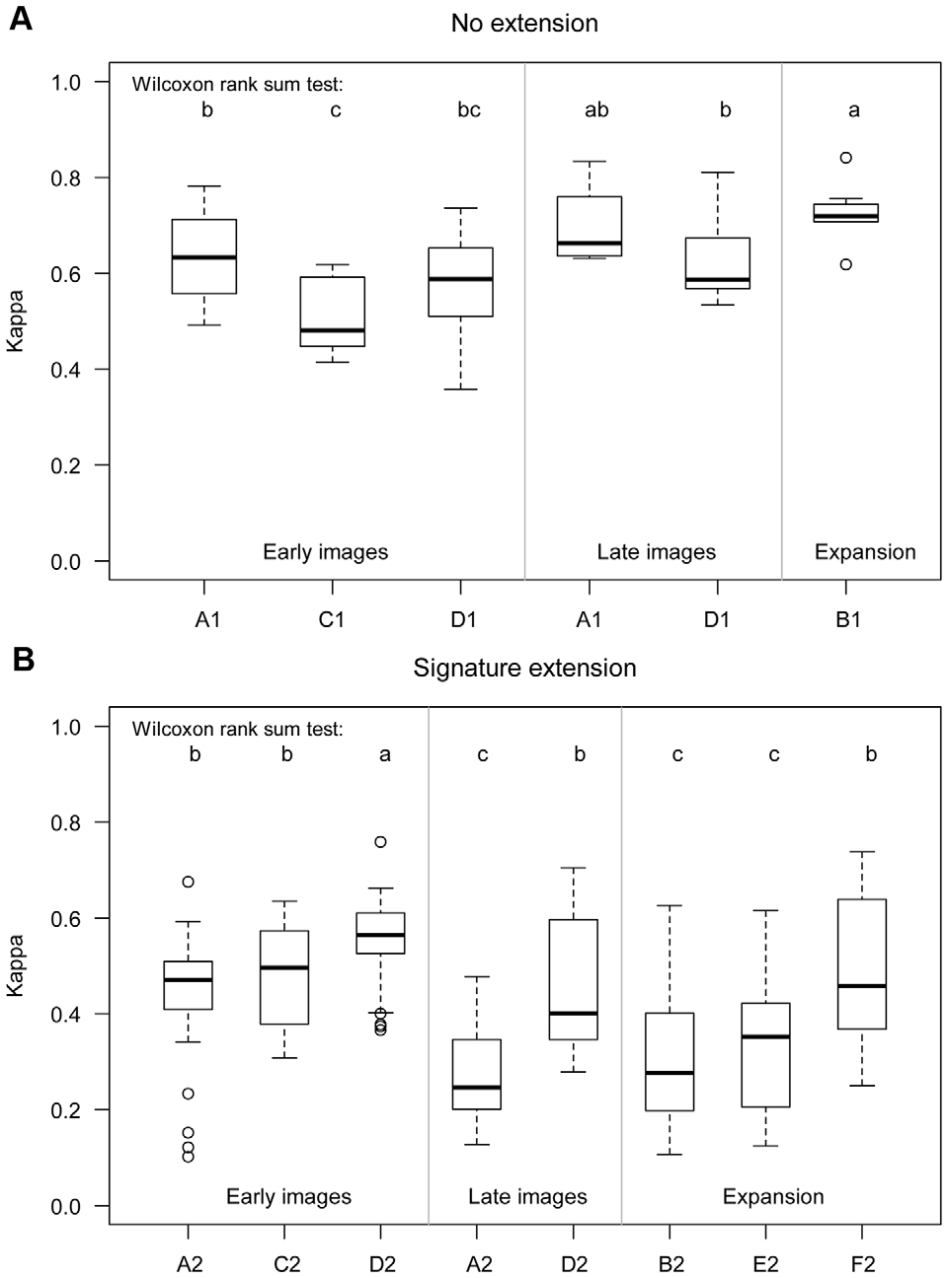
Classification accuracy for different methods of obtaining spectral signatures for land cover classes. Panels: A - no temporal extension, B - temporal extension; Methods: A (conventional), B (expansion), C (single-year generalization), D (multi-year generalization); and 1 (no temporal extension), 2 (temporal extension; see Figure 2). The boxes are from the first to the third quartiles and the line inside the box is the median. The whiskers extend to the most extreme data point or 1.5 times the inter-quartile range from the box, whichever is lower. Dots outside the whiskers are outliers. Methods with the same letter are not significantly different from each other (Wilcoxon rank sum test, α = 0.10).

Excluding classifications of images taken during the rainy season and considering all years included in the study, the average accuracy of conventional classifications of early images was not statistically different from that for late images (α = 0.1, Figure 5A A1). The average accuracy when combing these images (signature expansion, B1) was higher and less variable (mean = 0.73, standard deviation = 0.06) than the accuracies obtained with single-image classification of the early and late images. Although not statistically different from the conventional classification of the late images, the average accuracy resulting from signature expansion was statistically higher than that of conventional classification of early images.

#### Signature generalization

Signature generalization did not improve classification of images used in deriving the signatures. The mean accuracy of single-year generalization of early images declined by 19% compared with conventional classifications of early images (Figure 5A Early images C1 vs A1). Generalization of signatures using images from more than one year of early or late images was not statistically different from the corresponding conventional classifications (Figure 5A Early images D1 vs A1, Late images D1 vs A1). However, the standard deviation of the accuracy values obtained with signature generalization was reduced by more than one-third relative to that of conventional classification.

### Classification using signature extension

#### Conventional extension (using single image)

The commonly used method for signature extension, i.e., using a signature from a single image from another year, performed poorly compared with conventional classification. Average accuracy declined by 30% for early images and by 60% for late images (Figure 5A & B, A2 vs A1). Moreover, this method resulted in some extremely low accuracy values (Kappa <0.2). Among the lowest Kappa values observed (<0.2), half used the March 2005 image and the other half used a November image for deriving signatures.

#### Signature extension with generalization

With signature generalization involving images acquired from multiple years, signature extension led to significantly higher classification accuracy than conventional extension. This was the case for both early and late images (Figure 5B D2 vs A2). The classification accuracy from signature generalization of early images from only one year was not significantly different from that of conventional single-image extension (Figure 5B Early images C2 vs. A2).

Although late images usually had higher accuracy in conventional classifications, extension involving generalization resulted in higher accuracy for early images than for late images (Figure 5). Conventional extension in some cases resulted in extremely low accuracy values for either early or late images, whereas such low values were not observed in signature extension based on signature generalization.

**Figure 6:**
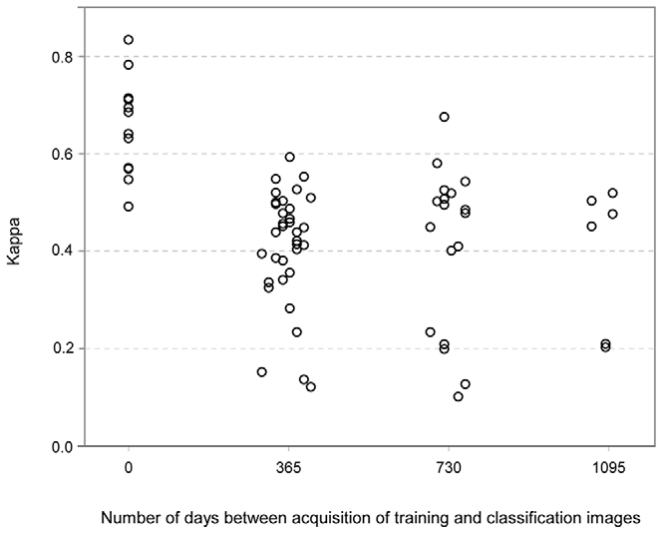
Classification accuracy and number of days between acquisition of training and classification images. Only pairs of images used for deriving signatures (training) and for classification (with and without signature extension) from the same season were included.

Accuracy was always lower with signature extension compared to conventional classification without signature extension, but, for same season comparisons, we did not find an association between accuracy and the number of days between acquisition dates of images used in training and in classification (Figure 6).

#### Signature extension with expansion

The combination of signature expansion and extension (B2) gave one of the worst average accuracies among all methods considered (i.e., second to A2, Figure 5B). With this method, the average classification accuracy was reduced by more than half and its standard deviation more than doubled compared to expansion without extension (Figure 5A & B, B2 vs B1).

#### Signature extension with generalization and expansion

Compared with signature extension with expansion (B2), the average accuracy did not improve with one-year generalization (E2). The mean accuracy was, however, significantly higher for generalization involving multiple years (F2). Moreover, multi-year generalization resulted in significantly higher accuracies compared with conventional signature extension using only late images (Late image A2) and did not result in extremely low classification accuracies as observed in conventional signature extension using only early images (Early image A2).

## Discussion

The large variation in accuracy of single-date image classifications suggests that even when no temporal signature extension or generalization is considered, it can be important to compare cross-seasonal images and select a single image or combinations of images that can be classified with high accuracy. Previous studies examining the use of multi-date Landsat images to classify land cover [12], [13] compared the classification accuracy of images from a single year (cropping season) only. Our study reveals that classification accuracy can strongly depend on the year and images used. In all four years, combinations of images had the highest accuracy (but the difference was sometimes small), but not all combined images had higher classification accuracy than the best of the single-date images. For example, in 2004, 5 out of 7 classifications involving expanded image signatures had lower accuracy than the November image classification for that year.

When temporal signature extension is considered, the use of signatures derived from a single-date image may result in classifications with an extremely low accuracy. To our knowledge, all previous studies dealing with temporal signature extension derived signatures from a single-date image from one year to classify an image from another year. We found that classification accuracy strongly depends on the image from which signatures are derived.

Our results also show that the drop in accuracy when using signature extension can be mitigated by signature generalization. Further research should investigate how general this finding is, but it is plausible that deriving signatures from multiple images can make the signatures more robust in the sense that they result in reasonably good classifications across years, but not necessarily produce the best classification in any single year. Robust signatures are needed for classification of time series of satellite images to monitor land cover change. The robustness of the generalized signatures is also illustrated by our finding that, in signature extension, the number of days between the dates of acquisition of images used for training and the dates of images used for classification did not affect accuracy, as was also found in [2].

Signature extension with generalization involving late images did not perform as well as signature extension involving early images. This was not expected, considering that the late images used in the generalization had a higher average accuracy under conventional classification. A possible explanation is that only one late image was available per year (i.e., December for 2005 and November for the other three years). Perhaps generalization could have resulted in higher accuracy had there been more than one late image per year as was the case for early images. The low accuracy observed with the combination of generalization and expansion could also be due to low number of late images available for the study area. Because there is only one late image and usually more early images available per year, the overall contribution of the late image is much higher because of duplicate occurrences resulting from the combination of expansion and generalization.

In comparing the effect on different land cover classes of signature expansion we expected that agriculture would benefit more than other classes as it has the strongest seasonal fluctuation of its reflectance characteristics. However this was not the case. We think this is because agriculture was easier to detect in earlier images (cleared fields) than in late images, when it becomes spectrally similar to shrubs, grasses and fallow land (the category for which classification accuracy improved most from signature expansion), and because we had very few usable growing season images (because of clouds).

It is possible that conventional signature extension performs better in other regions than in our study region. The stability of signatures derived from different combinations of training sites and images will depend, among other things, including atmospheric conditions during times that relevant images are acquired, and the crispness of the land cover classes considered. Our study was in a relatively difficult area for optical remote sensing of land cover, but these conditions are quite common across large geographic areas, particularly the tropical highlands. Further research in other regions should clarify this. We used a single algorithm to obtain spectral signatures. However, it could very well be that certain algorithms are better at creating more robust (less over-fitted) signatures than others, and this should also be addressed in research on the extension of spectral signatures.

The use of satellite images to study land cover over large areas and long time periods requires robust signatures. We described a number of methods, and a consistent nomenclature, to derive signatures by combining images within a year, across years, or both. Signature expansion, combining two images acquired during different seasons from the same year, often improved classification accuracy and reduced the variance in accuracy compared with conventional single-image classification. When signatures were extended to images from different years, the conventional approach performed poorly and multi-year signature generalization was more accurate. While our results may not be general for all areas, they clearly illustrate the need to carefully construct spectral signatures, and evaluate alternative approaches, including the derivation of a signature from several images, when classifying satellite images, particularly when applying a signature from one year to another.
